# Considerations in designing systems for large scale production of human cardiomyocytes from pluripotent stem cells

**DOI:** 10.1186/scrt401

**Published:** 2014-01-21

**Authors:** Allen Chen, Sherwin Ting, Jasmin Seow, Shaul Reuveny, Steve Oh

**Affiliations:** 1 Bioprocessing Technology Institute, 20 Biopolis Way, Centros #06-01, Singapore 138668, Singapore

## Abstract

Human pluripotent stem cell (hPSC)-derived cardiomyocytes have attracted attention as an unlimited source of cells for cardiac therapies. One of the factors to surmount to achieve this is the production of hPSC-derived cardiomyocytes at a commercial or clinical scale with economically and technically feasible platforms. Given the limited proliferation capacity of differentiated cardiomyocytes and the difficulties in isolating and culturing committed cardiac progenitors, the strategy for cardiomyocyte production would be biphasic, involving hPSC expansion to generate adequate cell numbers followed by differentiation to cardiomyocytes for specific applications. This review summarizes and discusses up-to-date two-dimensional cell culture, cell-aggregate and microcarrier-based platforms for hPSC expansion. Microcarrier-based platforms are shown to be the most suitable for up-scaled production of hPSCs. Subsequently, different platforms for directing hPSC differentiation to cardiomyocytes are discussed. Monolayer differentiation can be straightforward and highly efficient and embryoid body-based approaches are also yielding reasonable cardiomyocyte efficiencies, whereas microcarrier-based approaches are in their infancy but can also generate high cardiomyocyte yields. The optimal target is to establish an integrated scalable process that combines hPSC expansion and cardiomyocyte differentiation into a one unit operation. This review discuss key issues such as platform selection, bioprocess parameters, medium development, downstream processing and parameters that meet current good manufacturing practice standards.

## Introduction

Cardiovascular disease is the leading cause of death globally, accounting for 244.8 per 100,000 deaths in 2008 [1]. Although novel drugs and devices have enhanced the quality of life for patients with cardiovascular disease, they have not necessarily decreased morbidity or mortality [2]. Human adult cardiomyocytes have a turnover rate of less than 1% per year [3], indicating a limited regenerative capacity of the human adult heart. Resident cardiac stem cells and cardiac progenitor cells have been reported in the heart [4,5] and they have the ability to differentiate into all the constituent cell lineages of the myocardium, therefore participating in the repair process of a myocardial injury [6]. However, these cells cannot restore very large infarcts and an external therapeutic intervention is needed to compensate the heart’s inadequate intrinsic repair ability. As such, heart transplantation currently remains the only definitive treatment for end-stage patients. Unfortunately, donor hearts are critically deficient; thus, new therapeutic paradigms for heart failure are warranted.

A potential cure for heart failure can be achieved through cardiovascular cell therapy, which aims to repopulate damaged myocardium with new contractile cells and restore the heart. Pluripotent stem cells have nearly unlimited self-renewal capability *in vitro* and have the ability to differentiate into all three germ layers, thus giving rise to all cell types of the human body [7]. Since the initial demonstration that contracting cardiomyocytes can be generated from both human embryonic stem cells (hESCs) [8] and human induced pluripotent stem cells (hiPSCs) [9], stem cell technology has raised hopes for a source of unlimited numbers of human cardiomyocytes to rebuild the heart. In experimental animal models of acute myocardial infarction, transplantation of hESC-derived cardiomyocytes to the injury site has been shown to benefit heart function [10-12]. It was shown that the functional improvement of the heart is transient and presumably due to paracrine contributions of transplanted hESC-derived cardiomyocytes that led to increased vascularization [13]. Nevertheless, results presented so far are heartening because they present a prospect for survival and maturation of cardiomyocytes [14]. In cases of myocardial infarction, one billion cells potentially need to be replaced [15], emphasizing the need for reproducible and high yield differentiation protocols.

Besides their significance in regenerative medicine, cardiomyocytes generated *in vitro* are also needed for cardiac safety pharmacology testing. Unforeseen cardiotoxicity is one of the most common causes of late-stage clinical attrition [16]. Many drugs in the market have been withdrawn due to unexpected drug-induced electrophysiological alterations of the heart [17]. An example is the well-known case of rofecoxib, which was withdrawn from the market due to concerns about increased risk of cardiotoxicity and stroke associated with long-term, high dosage use. The early detection of any drug side effects can halt the process of futile and cost-intensive drug development and, more importantly, safeguard the health of patients. However, physiologically relevant *in vitro* cardiac models are limited as no current immortalized human cell lines accurately resemble functional cardiomyocytes of the heart for assessing preclinical cardiotoxic responses of drugs. Current cardiac models are typically animal models and *in vitro* assays, which lack cross-species translation due to differences in biological pathways and pharmacokinetic properties. Studies have already shown that hiPSC-derived cardiomyocytes will react to cardioactive drugs with expected responses, supporting the concept of large scale human cell-based predictive toxicology screens [18]. The use of human pluripotent stem cell (hPSC)-derived cardiomyocytes offers the pharmaceutical industry a precious tool for accelerating clinical introduction, reduce drug development costs and, most importantly, improving drug safety. Currently, GE Healthcare (GE Healthcare PTE Ltd., Singapore, Singapore) is providing cryopreserved hPSC-derived cardiomyocytes (Cytiva™ Cardiomyocyte). GE Healthcare has demonstrated that these cardiomyocytes can be used to develop a multiplexed cell imaging-based cardiotoxicity assay with fluorescent probes measuring plasma membrane integrity, cellular calcium levels, cell number and mitochondria status.

A third application of hPSC-derived cardiomyocytes is drug discovery. Over the years, many animal models, particularly mouse, have been generated and widely used. Although these models have shed light on our understanding of the onset and progression of cardiac diseases, they do not always recapitulate the phenotype seen in patients. Several studies have demonstrated the usage of hiPSC-derived cardiomyocytes as models for human cardiac disease [19-21]. Leveraging on models of cardiomyopathies, hPSC-derived cardiomyocytes can thus serve as a platform for screening existing therapies, testing experimental drug combinations and developing new ones. As high-content screens usually require more than 10^8^ purified cells [22], challenges to overcome are scalable differentiation methods and purification of cell fractions of interest.

The immense potential of hPSC-derived cardiomyocytes in regenerative medicine, cardiotoxicity pharmacology testing and drug discovery is indeed tantalizing. To effectively serve these applications, however, a reproducible, efficient and cost-effective platform for *in vitro* cardiomyocyte generation must be developed.

In this review, we examine the progress in development of large scale cardiomyocyte differentiation platforms from hPSCs. We describe the performance of existing reported systems and summarize the main obstacles for further research and optimization.

## Expansion of human pluripotent stem cells and cardiogenesis

The limited proliferating capacity of differentiated cardiomyocytes dictates the design of the production process. It is possible to isolate and cultivate cardiac progenitors as shown in several studies using fluorescence-activated cell sorting of the KDR+/PDGFRA- population [23] or genetically modified NKX2-5 reporter cell line [24]. Even though these progenitors have the potential to be used directly for cell therapy and as the source for cardiomyocyte generation [25], their isolation, maintenance requirements and expansion capacity provide several challenges. Wang and colleagues [26] have comprehensively reviewed the use of cardiac progenitors for cell therapy. Further, the differentiation step of hPSCs to cardiomyocytes has a limited cell expansion of two- to five-fold [27,28]. Thus, a scalable, large scale platform for cardiomyocyte production would have to emphasize hPSC expansion rather than cardiomyocyte expansion.

## Expansion of human pluripotent cells

Prior to establishment of a working cell bank and expansion method, it is important to select robust hPSC lines for growth ability and cardiomyocyte differentiation. Especially during the screening of hiPSC clones, it would be ideal to include parameters used in the later expansion as part of the selection criteria. For example, if microcarriers are chosen for hiPSC expansion, then suitable hiPSC clones should also be tested on microcarriers for cell growth and expression of pluripotent markers. In our group, we have observed variability in expansion capability of hPSCs as well as cardiomyocyte differentiation efficiency. Moreover, the mode of cell expansion (for example, feeder cells or extracellular matrix (ECM) coating) can affect cardiomyocyte differentiation efficiency. Hence, it is important to verify these effects before large scale cardiomyocyte generation.

**Figure 1:**
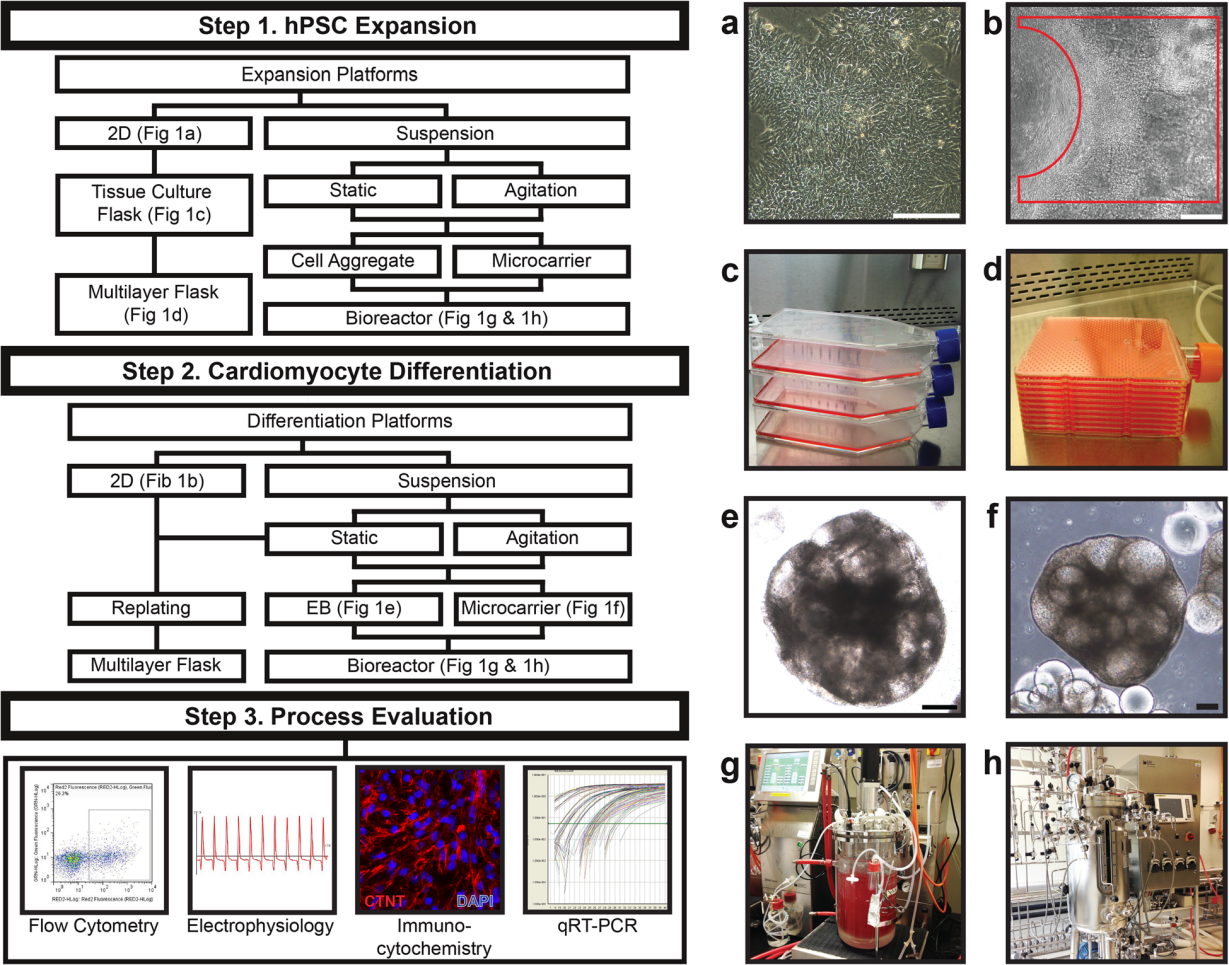
**Bioprocessing of human pluripotent stem cell-derived cardiomyocytes.** Illustrations showing phase contrast images of **(a)** two-dimensional human embryonic stem cell colony culture in mTeSR™1 medium and **(b)** human embryonic stem cell-derived beating cardiomyocytes (encircled by the red line). Scale bars are 200 μm. For small scale production, human pluripotent stem cells (hPSCs) can be expanded from **(c)** single layer tissue culture flasks to **(d)** multilayer flasks (Corning HYPERFlask^®^) with minimal controls. For clinical and commercial applications, hPSCs can be expanded and differentiated in suspension as **(e)** cell aggregates or on **(f)** microcarriers in bioreactors **(g,h)** with automated controls. 2D, two-dimensional; EB, embryoid body; qRT-PCR, quantitative reverse transcriptase-polymerase chain reaction.

As shown in Figure 1, three hPSC expansion platforms are available. To evaluate these different hPSC expansion platforms for cardiomyocyte generation, we will examine the technical issues involved in each and their implications on subsequent cardiomyocyte differentiation.

## Platforms for human pluripotent stem cell expansion

### Two-dimensional tissue culture

Conventional adherent monolayer culture has been widely utilized for hPSC expansion on either feeder cells or ECM (for example, Matrigel, BD Biosciences PTE Ltd., Singapore, Singapore) coated plates in conditioned medium or commercial serum-free medium such as mTeSR™1 (Stemcell Singapore PTE Ltd., Singapore, Singapore) and StemPro^®^hESC SFM (Life Technologies, Invitrogen Singapore PTE Ltd., Singapore, Singapore) (Figure 1a,c). The majority of reported cardiomyocyte differentiation protocols utilized either hPSCs co-cultured with feeder cells or on Matrigel with feeder cell-conditioned medium (Table 1). Expansion of hPSCs can be scaled up by using multi-layer flasks (Figure 1d). These multi-layer plates are currently utilized as the cell expansion platform according to good manufacturing practice for supporting cell therapy-related clinical trials [29]. Rowley and colleagues [29] estimated that up to 240 billion hPSCs can be produced with manual handling of 36 multilayer plates. For larger scales, suspension cultures were recommended (for example, microcarrier culture; step 1 in Figure 1g,h) [29]. Another drawback of hPSC expansion in these tissue culture flasks is the lack of representative sampling and online process monitoring or controls. This can undermine the consistency and the quality of the cells. Moreover, the production process requires extensive manual handling and large clean room facilities, which make large scale production expensive.

**Table 1:** Methods for cardiomyocyte differentiation from human pluripotent stem cells, ranked by purity of yields

**hPSC expansion platform**	**Cardiomyocyte differentiation platform**	**Biomolecules**	**Purity**	**Scalability**	**Comments**	**Reference**
Monolayer on feeders or Matrigel	Monolayer	Activin A, BMP4, VEGF, SCF, WNT3a	24% (Nkx 2.5)	+		[25]
		BMP2, 5% FBS	41.6% (cTnT)	+		[30]
		Activin A, BMP4	51% (MHC)	+		[10]
		Activin A, BMP4, DKK1	54.2% (cTnT)	+		[31]
		Activin A, BMP4, FGF2 , VEGF, DKK1	57.2% (cTnT)	+		[32]
		Activin A, BMP4	60% (α-actinin)	+		[33]
		Activin A, BMP4, IWP-4 or IWR-1	60.6% (MHC)	+		[34]
		Activin A, BMP4, FGF2, Noggin, BMS-189453, DKK1	73.0% (cTnT)	+		[35]
		BMP4, ascorbic acid, CHIR99021, IWR-1	80% (cTnT)	+		[36]
		Activin A, BMP4, FGF2	80% (cTnT)	+		[27]
		Activin A, BMP4, VEGF	85.4% (cTnT)	+		[37]
		CHIR99021, IWP-2/IWP-4	88.3% (cTnT)	+		[38]
		FBS	90% (cTnT)	+		[39]
		CHIR99021, BIO, KY02111, XAV939	97.7% (cTnT)	+		[40]
	Embyoid bodies	Activin A, FGF2	23.6% (beating EBs)	++	Forced aggregation	[41]
		Normoxic	48.3% (beating EBs)	++	Micropatterned; controlled bioreactor	[42]
		BMP4	95.8% (beating EBs)	+	Replated after 4 days	[43]
		Ascorbic acid	6.94% (cTnT)	+	Replated after 5 days	[44]
		BMP4, IWP-1	15.6% (cTnT)	+	Replated after 4 days	[45]
		SB203580	16% (MHC)	++		[28]
		SB203580	22.0% (MHC)	++		[46]
		BMP2, 5-azacytidine	23.7% (cTnT)	+	Replated after 6 days	[47]
		SB203580	26% (MHC)	++		[48]
		Activin A, BMP4, FGF2, VEGF, SCF	26.8% (Nkx 2.5)	++	Forced aggregation	[24]
		Activin A, BMP4, FGF2, VEGF	27.1% (MHC)	+	Replated after 4 days	[49]
		Activin A, BMP4, FGF2, VEGF, DKK1	37.2% (cTnT)	++		[50]
		WNT3a	50% (α-actinin)	+	Replated after 6 days	[51]
		BMP2	53.3% (cTnT)	++		[52]
		CHIR99021, IWP-2	60% (cTnT)	++		Unpublished data
		Activin A, BMP4, FGF2, VEGF, DKK1	60.2% (cTnI)	++		[53]
		Activin A, BMP4, FGF2 , VEGF, DKK1	82% (cTnT)	++		[23]
		BMP4,FGF2	82.3% (cTnI)	++	Forced aggregation	[54]
		Activin A, BMP4, FGF2, VEGF,DKK1	91.6% (cTnT)	++	Dissociated EBs	[55]
	Microcarriers	SB203580	20% (MHC)	+++		[28]
Cell aggregate	Monolayer	Activin A, BMP4, FGF2	80% (cTnT)	+		[27]
	Embryoid bodies	Activin A, BMP4, FGF2, VEGF, DKK1	27% (cTnT)	+++		[56]
		BMP4	36.9% (Nkx 2.5)	+	hESC encapsulation	[57]
		Activin A, BMP4, FGF2, VEGF, IWR-1	80% (cTnT)	+++	Controlled bioreactor	[27]
	Microcarriers	No data				
Microcarriers	Microcarriers	CHIR99021, IWP-2	67% (cTnT)	+++		Unpublished data

*BMP*, bone morphogenetic protein; *cTnI*, cardiac troponin I; *cTnT*, cardiac troponin T; *EB*, embryoid body; *FBS*, fetal bovine serum; *FGF*, fibroblast growth factor; *hESC*, human embryonic stem cell; *hPSC*, human pluripotent stem cell; *MHC*, myosin heavy chain; *SCF*, stem cell factor; *VEGF*, vascular endothelial growth factor.

### Cell aggregate culture

Cell aggregate culture has been developed as a scalable expansion platform for hPSCs, which utilizes the characteristic of hPSCs to form aggregates in suspension. hPSCs in single cell suspension usually undergo apoptosis or anoikis. In order to prevent these detrimental effects and establish a viable hPSC suspension culture, hPSCs are partially dissociated in order to form cell aggregates and/or ROCK-Myosin signaling pathway inhibitors (Y27632 or Blebbistatin) are added to improve survival of dissociated hPSCs. Despite significant cell loss during the initial cell aggregate formation (28 to 76%), several groups have obtained cell concentrations of 1 to 2 × 10^6^ cells/ml with 12.5- to 17.7-fold expansion using various hPSC lines [56,58-60]. Since hPSC aggregation is usually associated with spontaneous differentiation, maintaining pluripotency can be demanding. Excessive cell aggregation can induce spontaneous differentiation or necrosis due to nutrient diffusion limitation. The maintenance of aggregate culture is usually carried out by limiting aggregate size to under 500 μm by constant disaggregation of the culture. The process is done manually and can affect culture viability and reproducibility. Designing a scalable bioprocess for aggregate culture may require the integration of reproducible, automated processes for repetitive dissociation and formation of uniform cell aggregates. hPSC aggregate cultures were recently reviewed by O'Brien and Laslett [61].

### Microcarrier culture

The development of the microcarrier platform for hPSC expansion has attracted interest for generating cardiomyocytes in a scalable manner. hPSCs cultured on microcarriers exhibited higher cell density and expansion than two-dimensional tissue or aggregate cultures, while maintaining pluripotency and karyotypic stability [62]. A recent study by Bardy and colleagues [63] reported the highest cell concentration of 6.1 × 10^6^ cells/ml with 20-fold expansion in spinner flasks in serum-free medium. The microcarrier-expanded cells exhibited the capacity to differentiate *in situ* to neural progenitors [63], endoderm progeny [64], and cardiomyocytes [62,65]. However, in order to establish a viable hPSC microcarrier culture, several factors have to be considered, such as microcarrier coating and type, bioreactor design, operating parameters and feeding of medium. These factors can have significant impacts on cell growth, pluripotency and lineage commitments [66]. Compared to other classical microcarrier cultures (for example, Vero cells and mesenchymal stem cells) that form monolayers of cells on microcarriers, hPSCs form multi-layered cell-microcarrier aggregates during propagation. Several groups have screened a variety of microcarriers and coatings for initial cell attachment and hPSC expansion and examined their impact on prolonged cultivation [64,65,67,68]. A study by Chen and colleagues [67] examined in detail the effects of shape, size, surface charge, and coatings of microcarriers on hPSC cultivation. Microcarrier shape and size affect the compactness of cell-microcarrier aggregates and consequently cell growth. hPSCs cultured on spherical microcarriers (Ø >180 μm) exhibited more open structures whereas compact cell-microcarrier aggregates were observed in cylindrical microcarriers [67]. The compactness of cell-microcarrier aggregates increased with the decrease in microcarrier diameter and this had a negative impact on cell growth, likely due to limited nutrient diffusion for the cells within the aggregates [67]. Matrigel coating has been shown to be critical for hPSC cultivation on microcarriers. Recently, it was shown that laminin and vitronectin can replace Matrigel as defined coatings on microcarriers without compromising cell growth [69]. ROCK inhibitors (Y27632 or Blebbistatin) were later shown to enable hPSC growth on ECM coating-free microcarriers [70].

Scaling up of hPSC microcarrier cultures has been demonstrated by several groups using spinner flasks. Serra and colleagues [71] carried out hPSC microcarrier culturing in bioreactors (300 ml) with automated process control. The authors achieved 2.17 × 10^6^ cells/ml in 11 days with a seeding density of 1.5 × 10^5^ cells/ml (15-fold expansion). A comprehensive review of the bioprocess parameters for hPSC microcarrier culture was recently published [66]. It is important to note that hPSCs propagated on microcarriers can undergo changes in their phenotype. hPSCs cultured on microcarriers exhibited longer doubling time and a generally lower lactic acid specific production rate than those cultured on a tissue culture plate in serum-free media [72]. Leung and colleagues [73] reported different sensitivities of hPSC lines to shear stress. hPSCs expanded on microcarriers in spinner flasks were able to differentiate efficiently to neural progenitors [63] and endoderm progeny [64]. However, this was not the case for cardiac differentiation. We have observed that hPSCs expanded in agitated microcarrier cultures and differentiated to cardiomyocytes by the p38 MAP kinase protocol [28,74] exhibited lower yields of cardiomyocytes when compared to cells expanded in static microcarrier culture (unpublished results). We hypothesized that the shear effect caused subtle changes in hPSCs that reduced their cardiac differentiation propensity. Hence, it is important to establish quality controls to monitor these subtle changes in hPSC culture before cardiac differentiation.

### Cardiogenesis

**Figure 2:**
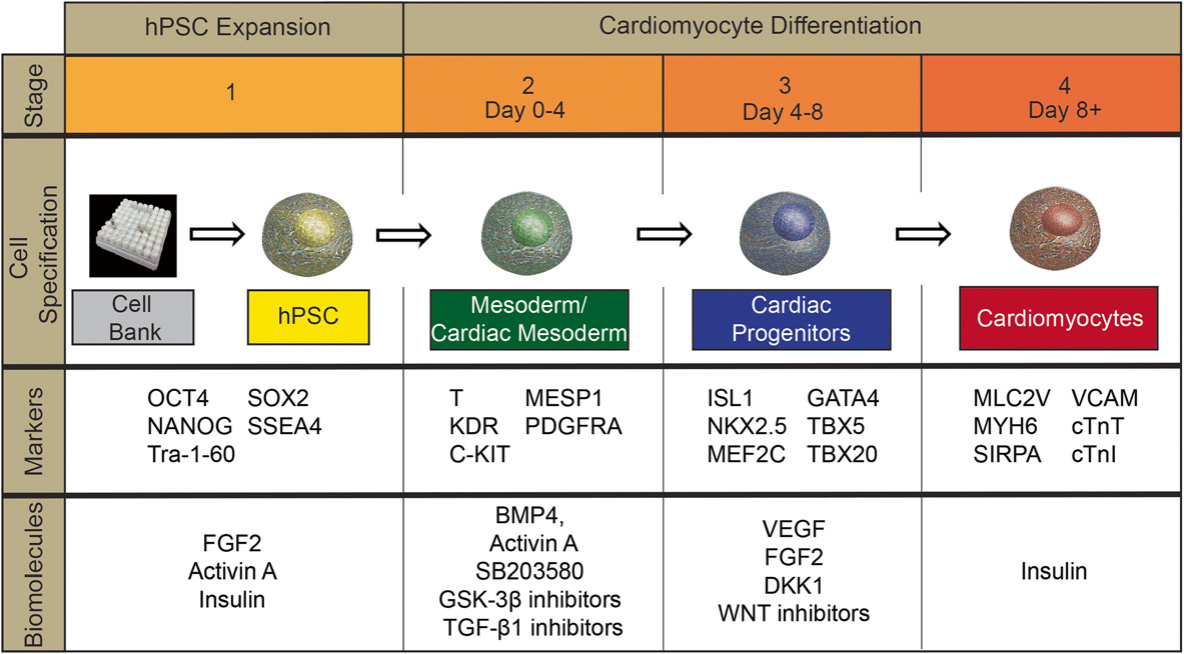
**Schematic of human pluripotent stem cell expansion and differentiation to cardiomyocytes.** Biomolecules (growth factors and small molecules) play important roles in human pluripotent stem cell (hPSC) expansion and differentiation to cardiomyocytes. Intracellular and cell surface markers associated with each of the three main stages can be used to monitor the progression of differentiation.

Cardiomyocyte differentiation is based on the recapitulation of *in vivo* cardiogenesis, which depends on a series of complex molecular signaling pathways [75,76]. The differentiation efficiency of these methods depends on the biomolecules used (growth factors or small molecule inhibitors), hPSC culture expansion conditions and the timely activation or deactivation of molecular signals necessary to guide the differentiation toward cardiac lineages (Figure 2) [77]. Moreover, in all protocols, the concentrations and duration of growth factors or small molecule inhibitor treatments depend on the platform (embryoid body (EB), two-dimensional tissue culture or microcarrier cultures) and the hPSC line.

Current cardiac differentiation protocols use either small molecule inhibitors or growth factors to induce the signals for cardiac differentiation. In both cases, the signaling leads to a cascade of three sequential stages: mesodermal induction, cardiac progenitor and cardiomyocyte generation and maintenance [78] (Figure 2). Mesodermal induction, which is monitored by the expression levels of KDR and PDGFR-α [23,53], takes place during the first 3 to 4 days of cardiomyocyte differentiation. This step is usually induced with the growth factors, bone morphogenetic protein (BMP)4 and Activin A, which activate the transforming growth factor (TGF)-β signaling pathway, which is crucial for mesoderm differentiation [79]. Mesoderm induction can be also achieved by the addition of small molecules, such as GSK-3β inhibitors (CHIR99021 or BIO) [38,40]. These inhibitors increase endogenous levels of BMP2/4, thus indirectly activating the TGF-β signaling pathway [38].

Cardiac progenitor induction is achieved by removal of the TGF-β pathway activators and addition of the growth factors, fibroblast growth factor-2 and/or vascular endothelial growth factor, which activate the ERK signaling pathway [80], or by small molecules that inhibit WNT signaling (for example, KY02111, XAV939, IWP-2 and IWR-1). As these factors drive mesodermal cells towards the cardiac progenitor lineage, they inhibit the development of smooth muscle and endothelial cell lineages [32,50]. A common finding in this stage is that the addition of insulin inhibits the cardiac progenitor differentiation process [49].

The final stage of cardiomyocyte production (day 8 and later) focuses on maturation of cells to cardiomyocytes and their maintenance. Differentiated cardiomyocytes can be maintained in simple serum-free medium, which can minimize outgrowth of fibroblasts and maintain cardiomyocyte purity. Prolonged cultivation of cardiomyocytes has been shown to increase the amount of mature ventricular phenotype [38].

## Current platforms for generating cardiomyocytes from human pluripotent stem cells

After having established a scalable hPSC expansion method, a suitable cardiac differentiation process should be developed. The variety of protocols used by research groups indicates the complexity of the differentiation process and hence the difficulty of applying universally efficient differentiation protocols to the different hPSC lines and culture conditions (Table 1). To date, cardiomyocyte differentiation platforms can be divided into three categories, namely monolayer, EB and microcarrier cultures. Selecting a suitable platform for cardiomyocyte production may depend on several factors, including type of intended application (for example, cell therapy, disease modeling, cardiac toxicology study).

### Two-dimensional tissue culture

In the two-dimensional tissue culture differentiation platform, hPSCs expanded in monolayer can be directly differentiated to cardiomyocytes by a simple change of hPSC growth medium to cardiac differentiation medium. The first efficient directed differentiation protocol was reported by co-culturing hESCs with mouse endoderm-like cells (END-2), which generated 85% cells exhibiting ventricular-like action potentials [81]. Differentiation protocols were further refined by using more defined conditions with known growth factors, extracellular matrices and eliminating the need for END-2 cells. As shown in Table 1, a simple protocol of using serum-free medium supplemented with Activin A and BMP4 can direct the differentiation to cardiomyocytes. In brief, Activin A was added to confluent hPSC monolayer culture for 1 day, then BMP4 for 4 days followed by the removal of growth factors and maintenance in serum-free medium afterward [10]. The protocol was further improved with additional growth factors or small molecules to accommodate interline variability that usually exists among hPSC lines. Recently, small molecules targeting TGF-β, BMP and WNT signaling have shown the potential to replace growth factors in directing hPSC to cardiomyocytes [38]. In particular, small molecules targeting WNT signaling were the most promising. The sequential activation of WNT via GSK-3β inhibitors (for example, CHIR99021 and BIO) for 1 day, followed by WNT inhibition (for example, KY02111, XAV939, IWP-2 and IWR-1) at day 3 and removal of small molecules after day 5 achieved cardiomyocyte purity of up to 98% at day 14 [38]. Prolonged cultivation or using the novel WNT inhibitor KY02111 generated more ventricular cardiomyocytes [38,40]. These molecules have been applied successfully in cardiomyocyte differentiation using hPSCs cultured on synthetic ECM peptide coated plates [38].

### Embryoid body culture

In the EB differentiation platform, cells from two-dimensional cell cultures are usually dissociated into single cells followed by re-aggregation in order to generate homogeneous EB cultures with uniform sizes that are further differentiated to cardiomyocytes (Figure 1e). In general, this dissociation process can lead to significant cell death [82]. Differentiating hPSCs as EBs was first employed in order to accurately recapitulate the complex assembly of cell adhesion and intracellular signaling of early embryogenesis [82]. hPSCs undergo spontaneous differentiation when cultured in suspension as EBs, forming cells of the three lineages. Without guidance towards cardiomyocyte differentiation and control of the culture conditions, the efficiency of cardiomyocyte differentiation was usually low (<1%) [30].

Significant progress has been made to improve EB-based cardiomyocyte differentiation efficiency with the identification of growth factors and pathways associated with cardiac differentiation (Table 1). Most of the reported EB cultures use hPSCs expanded in two-dimensional tissue cultures on feeder cells, in particular mouse embryonic fibroblasts. Upon cell dissociation, the residual feeder cells in the cell suspension provide attachment matrices for the generation of EBs. These EBs achieve higher cell viability and aggregate stability compared to EBs generated from two-dimensional tissue cultures without feeder layers (for example, Matrigel) [83].

EB differentiation protocols are more complicated than those used in two-dimensional tissue cultures due to their three-dimensional shape and the effects of the EB microenvironment [82]. Specifically, the size of EBs has been shown to influence cell differentiation towards different lineages [84]. In addition, concentrations of small molecules or growth factors used for differentiation had to be adjusted as these soluble factors have to diffuse through a multi-layered cell environment [82]. As cells differentiate toward the cardiac lineage, the formation of excessive aggregate size as a result of cell proliferation or attachment of several aggregates into one (agglomeration) could cause necrosis and consequently reduce cardiomyocyte yield [74]. In order to overcome these problems, several groups have placed single dissociated cells into V- or U-bottom micro-wells coupled with centrifugation to generate uniform cell-aggregates with cell numbers ranging from 200 to 1,000 cells per aggregate [53,58]. Others have tried encapsulation of hESCs and differentiation to cardiomyocytes [57]. We have tested the use of inert dextran beads as a means to separate cell aggregates from each other. In brief, dextran beads were added to culture wells containing EBs to fill spaces between aggregates, forming two to three layers of beads. Without these inert beads separating the aggregates, frequent manual manipulation of cell aggregates is required in order to prevent agglomeration [74]. In some cases, 4-day-old EBs can be plated on ECM-coated tissue culture plates (for example, gelatine), forming beating cell layers afterward. Under these culture conditions, the yield and purity of the cardiomyocytes improved significantly, achieving 64 to 89% cardiac troponin T (cTnT)-positive cardiomyocytes with a variety of hPSC lines [54].

Furthermore, hPSCs can be expanded as cell aggregates in suspension without microcarriers as discussed previously. Two studies showed that these aggregate cultures expanded in a spinner flask can be subsequently differentiated *in situ* to cardiomyocytes as EBs [56,58]. Matsuura and colleagues [27] reported 80% cTnT-positive cardiomyocytes generated in a controlled bioreactor. Even though the efficiency of cardiomyocyte differentiation was lower than some of the reported EB-based methods (Table 1), cell aggregate expansion followed by *in situ* differentiation is more scalable than monolayer expansion.

### Microcarrier platform

Microcarriers can be applied in two ways for cardiomyocyte differentiation. Firstly, they can be used to assist EB formation, stabilization and prevention of agglomeration. Lecina and colleagues [28] used five different microcarriers to examine the effect of microcarrier type, size, shape and concentration on the efficiency of EB-based cardiomyocyte differentiation using SB203580, a mitogen-activated protein kinase inhibitor. Only the small positively charged Tosoh-10 beads of Ø10 μm, which did not support cell expansion [67], were able to stabilize EB structures and achieve efficient cardiomyocyte differentiation. Using these microcarriers, a cardiomyocyte yield of 90% beating aggregates and 17% cells expressing cardiomyocyte markers (myosin heavy chain and alpha-smooth muscle actin) were achieved in agitated cultures. In these cultures, two- to three-fold higher efficiency (0.28 to 0.62 cardiomyocytes generated per hESC seeded) than the EB-based method (0.13 to 0.22 cardiomyocyte generated per hESC seeded) was achieved [28,74]. Conventional larger microcarriers (Cytodex1 (Ø190 μm) and DE-53 (length 130 μm × diameter 35 μm)) that exhibited higher hPSC expansion capability were not efficient in generation of beating cell-microcarrier aggregates during the cardiomyocyte differentiation process.

In a second approach, hPSC microcarrier aggregates generated during cell expansion can be directly differentiated to cardiomyocytes [62,65]. Reported experimental results using this approach are still limited. However, our group has applied microcarrier expanded hPSC cells for cardiomyocyte differentiation and obtained high cell density of 8 × 10^6^ cells/ml (40.4-fold cell expansion) with high cardiomyocyte purity of 67% cTnT and yields of 268 cardiomyocytes per hPSC seeded (Figure 1f; unpublished data). Furthermore, we have observed that the method of hPSC microcarrier culture expansion (static versus agitated condition in spinner flask) can affect the efficiency of cardiomyocyte differentiation. The cause for this phenomenon is still under investigation.

## Considerations for scaling up cardiomyocyte production

We have analyzed three hPSC expansion platforms (two-dimensional, cell-aggregate and microcarrier cultures) for their potential to be coupled with various differentiation platforms. These platforms can be applied using a variety of media, culture methods and conditions. In the following section we discuss critical criteria that should be considered in selection of conditions for designing a scalable cardiomyocyte production system.

### Selection of cardiomyocyte production platform

The selection of cardiomyocyte production platform is dictated by the quantity of cardiomyocytes required for a particular application. Three methods of hPSC expansion and seven options for cardiomyocyte differentiation are available for cardiomyocyte production (Table 1). The initial selection of hPSC expansion platform would be based on the amount of cardiomyocytes and the purity needed. The monolayer culture platform can be considered the most straightforward method when compared to suspension-based systems, achieving relatively high differentiation efficiency (Table 1). For large scale production purposes, cell-aggregate and microcarrier-based systems should be considered. Furthermore, process monitoring and control can be easily applied in suspension platforms to ensure process consistency and reproducibility (Figure 1g,h). We believe the integration of hPSC expansion and cardiomyocyte differentiation as a one unit operation in suspension would be the best approach to scale up cardiomyocyte production.

### Bioprocess parameters for cardiomyocyte production in suspension

hPSC expansion in suspension with automated online process monitoring and controls has been reported [71]. Serra and colleagues [71] examined the effects of oxygen and operation mode (perfusion versus semi-continuous) in hPSC microcarrier cultures [71]. Oxygen was preferable at 30% air saturation instead of 5%. hPSCs in perfusion mode (dilution rate of 0.5 day^-1^) showed lower lactate production and shorter lag phase than semi-continuous culture (50% medium exchange per day) [71]. The perfusion system might be ideal for stage-specific cardiomyocyte differentiation compared to current processes with constant fluctuations in metabolite and growth factor concentrations. Such a system with optimized medium feed can also provide greater efficiencies, replacing current empirical media feeding schemes for hPSC expansion and cardiomyocyte differentiation.

Maintaining a homogeneous suspension culture is critical in process monitoring and controls, but can be challenging considering the size variations of cell aggregate and cell-microcarrier aggregate cultures. Stirring can also induce shear stress responses. Recently, we have observed that agitation-induced shear stress in spinner microcarrier cultures reduced cardiomyocyte differentiation efficiency. Applying agitation during the first 3 days of cardiomyocyte differentiation (for both microcarrier- and EB-based methods) suppressed cardiomyocyte differentiation. This inhibition could be alleviated when intermittent agitation was employed. The literature reports that shear stress affects the TGF-β pathway, which is vital in cardiomyocyte differentiation [85,86].

### Medium development

Medium development is critical in the development of hPSC expansion and differentiation processes. In most papers, hPSCs were expanded in conditioned medium on Matrigel-coated plates or mitotically inactivated feeder cells (Table 1). Medium components like serum replacement (Knockout Serum Replacement (KOSR, Life Technologies)), feeder cells and Matrigel for coating of plates or microcarriers pose risks of pathogen contamination. Therefore, defined xeno-free conditions for hPSC expansion have been developed. Lately, it was reported that hPSCs can be cultured in a simple xeno-free, defined medium with eight components on recombinant vitronectin coated plates [87]. This defined and cheaper medium has significant impact on future hPSC clinical applications where the high medium cost has been one of the bottlenecks for studying large scale process development. This medium can also be adapted for hPSC expansion in aggregate or microcarrier cultures. Significant progress has also been made in establishing defined media for each stage of cardiomyocyte differentiation. Media used by various groups are relatively defined and include known growth factors or small molecules with trace amounts of serum albumin (bovine or human) added. Serum-supplemented medium has been used by several groups in generating stable EBs (Table 1). To tackle this issue, Ng and colleagues [88] developed a defined medium, denoted as APEL, for EB formation and differentiation. To address the cost efficiency, small molecules that target WNT signaling transduction pathways have been found to be suitable replacements for costly growth factors.

### Downstream processing

Studies on development and optimization of downstream processing of hPSC-derived cardiomyocytes are very limited. The process of harvesting cardiomyocytes from monolayer, EB or microcarrier cultures has been demonstrated for small scale research purposes. Monolayer cultures, EBs or cell-microcarrier aggregates are treated with dissociation enzymes followed by passing through a sieve to remove undissociated cell aggregates and microcarriers [74]. These processes are usually not optimized and can become more problematic during scaling up. Enzyme concentration, treatment time, mode of mixing, and sieve loading capacity can affect cell viability and harvest efficiency. Hence, there is a need for further investigation into establishing a scalable and efficient cell harvesting process.

Another aspect of downstream processing is the purification of cardiomyocytes from mixed cell populations [31,89]. Considering the amount of cells required for cell therapy, the effects of unwanted cells in cell replacement therapy are largely unknown. Tumorigenicity related to undifferentiated hPSCs remains one of the main concerns, which can be addressed by two approaches. Choo and colleagues [90] used cytotoxic monoclonal antibodies against hPSCs to eliminate undifferentiated hPSCs. Others reported the use of magnetic activated micro-beads coupled to multiple antibodies to remove undifferentiated hPSCs and enrich cardiomyocytes from the heterogeneous cell population after differentiation [31,89,91,92]. However, the antibody-based approach might not be economically viable when scaled up due to the quantity of antibodies needed and their associated costs. Alternatively, Tohyama and colleagues [93] showed a novel approach based on cardiomyocyte metabolic properties, in which cardiomyocytes (up to 99% purity) were selected from mixed cell populations using glucose-depleted medium containing high lactate. This method is inexpensive, scalable and could be easily integrated into a cardiomyocyte production platform. In addition, hPSC-derived cardiomyocyte cultures contain three sub-phenotypes (atrial, ventricular and nodal). The ratio between these subtypes can be altered by modulating signaling pathways during the differentiation [78]. It was reported that activation of neuregulin/ErbB signaling can increase the nodal fraction within the cardiomyocyte population [94]. Overexpression of microRNA 499, decreased retinoid signaling, electrical stimulation, prolonged cultivation and use of the WNT inhibitor KY02111 were shown to increase the ventricular subtype [35,38,40,95,96]. Thus, isolation and purification of the required sub-phenotype could be further investigated in order to improve efficiency. Specifically, enriched nodal cells could potentially form a biological pacemaker whereas ventricular cardiomyocytes could be used for cell therapy after ventricular myocardial infarction.

Lastly, the purified cardiomyocytes can be either cryopreserved or applied directly for tissue engineering. Progress has been made showing the functionality of cryopreserved and dissociated cardiomyocytes. A study by Xu and colleagues [97] showed that cardiomyocytes can be dissociated into single cells, cryopreserved and thawed without significant loss (70-77% recovery). These cells could be used for transplantation, comparable to freshly isolated cells [97].

### Meeting current good manufacturing practice requirements

Production of cardiomyocytes at clinical commercial scales that follows the current good manufacturing practice (cGMP) standard will be challenging as most of the current processes are carried out for research purposes without any consideration for cGMP requirements. A defined xeno-free integrated process of expansion, differentiation and downstream purification within a closed system with appropriate monitoring and control systems would be the most suitable for cGMP manufacturing. To characterize the cardiomyocytes produced, quality control assays (step 3 in Figure 1) should be standardized to include cardiomyocyte gene (for example, quantitative RT-PCR) and protein marker (flow cytometry and immunocytochemistry) expression analysis, characterization of electrophysiological properties (multi-electrode array and patch clamp), structural property and organization (electron microscopy) analysis, and determination of calcium signaling (calcium imaging) [98]. Automated high-throughput imaging-based assays developed recently can be advantageous for providing reliable, non-invasive, multi-parameter, real-time monitoring of cardiomyocytes in suspension as EBs or on microcarriers. In cases where downstream purification of cardiomyocytes to subtypes is performed, quality control of cell identity should be done by using the whole cell patch clamping technique, high content immunocytochemistry and flow cytometry [78]. Patch clamping measures the action potential of individual cells and can be both time consuming and low throughput. Flow cytometry analysis using antibodies against these subtypes has been utilized as a high-throughput method. Antibodies against myosin light chain 2 (MLC2) isoforms, namely MLC2a and MLC2v, are widely used to determine ventricular and atrial-like cardiomyocytes.

## Conclusions and future directions

Cardiac differentiation is an extremely delicate and dynamic process that involves the activation and inhibition of multiple signaling pathways at different time points. Due to this complexity, the development of a protocol that can efficiently differentiate hPSCs to cardiomyocytes in a scalable platform has still yet to be developed and optimized. In this review, current platforms for hPSC expansion have been reviewed for their propensity to be adapted into a scalable bioprocess and their efficiency in terms of cardiomyocyte differentiation. The different protocols developed over the past few years have focused on generation of high purity cardiomyocytes without considering issues involved in scaling up the processes. Establishment of a scalable cardiomyocyte production platform requires a more holistic approach integrating parameters related to scaling up hPSC expansion, cardiomyocyte differentiation and downstream purification into a one unit operation.

In summary, current demands from regenerative medicine, drug testing, and disease modeling require development of cardiomyocyte production processes that have to meet a variety of requirements (for example, serum-free media, cGMP requirements, cost of production, quality control and downstream bioprocessing). Beyond these issues, several others have to be considered in future applications. For example, cardiomyocytes produced according to the current methods are immature in their marker expression and electrical and mechanical functionality [77]. This poses problems for the applications mentioned previously as immature cardiomyocytes may not be the most ideal models of adult cardiomyocytes. Moreover, for cell therapy an appropriate cell delivery method should be developed [77,99]. These challenges call for multidisciplinary efforts that adapt current cardiomyocyte differentiation protocols to develop a cost-effective, scalable and cGMP compliant process, and that resolve issues of downstream purification and quality control as well as cell maturation and delivery systems.

### Note

This article is part of a thematic series on *Cardiovascular regeneration* edited by Ronald Li. Other articles in the series can be found online at http://stemcellres.com/series/cardiovascular

## Abbreviations

BMP: Bone morphogenetic protein; cGMP: current good manufacturing practice; cTnT: cardiac troponin T; EB: Embryoid body; ECM: Extracellular matrix; hESC: human embryonic stem cell; hiPSC: human induced pluripotent stem cell; hPSC: human pluripotent stem cell; MLC2: Myosin light chain 2; RT-PCR: Reverse transcriptase-polymerase chain reaction; TGF: Transforming growth factor.

## Competing interests

The authors declare that they have no competing interests.
